# Recent advances in non-small cell lung cancer targeted therapy; an update review

**DOI:** 10.1186/s12935-023-02990-y

**Published:** 2023-08-11

**Authors:** Mahmood Araghi, Reza Mannani, Ali Heidarnejad maleki, Adel Hamidi, Samaneh Rostami, Salar Hozhabri Safa, Fatemeh Faramarzi, Sahar Khorasani, Mina Alimohammadi, Safa Tahmasebi, Reza Akhavan-Sigari

**Affiliations:** 1 Department of Pathology, School of Medicine, Zanjan University of Medical Sciences, Zanjan, Iran; 2 Vascular Surgeon, Department of Surgery, Faculty of Medicine, Zanjan University of Medical Sciences, Zanjan, Iran; 3 School of Medicine, Zanjan University of Medical Sciences, Zanjan, Iran; 4 Razi Vaccine and Serum Research Institute, Arak Branch, karaj, Iran; 5 Department of Immunology, School of Medicine, Mazandaran University of Medical Sciences, Sari, Iran; 6 Department of Immunology, School of Public Health, Tehran University of Medical Sciences, Tehran, Iran; 7 Department of Immunology, School of Medicine, Shahid Beheshti University of Medical Sciences, Tehran, Iran; 8 Student Research Committee, Department of Immunology, School of Medicine, Shahid Beheshti University of Medical Sciences, Tehran, Iran; 9 Department of Neurosurgery, University Medical Center, Tuebingen, Germany; 10 Department of Health Care Management and Clinical Research, Collegium Humanum Warsaw Management University Warsaw, Warsaw, Poland

**Keywords:** NSCLC, Targeted therapy, Target antigens, Immunotherapy

## Abstract

Lung cancer continues to be the leading cause of cancer-related death worldwide. In the last decade, significant advancements in the diagnosis and treatment of lung cancer, particularly NSCLC, have been achieved with the help of molecular translational research. Among the hopeful breakthroughs in therapeutic approaches, advances in targeted therapy have brought the most successful outcomes in NSCLC treatment. In targeted therapy, antagonists target the specific genes, proteins, or the microenvironment of tumors supporting cancer growth and survival. Indeed, cancer can be managed by blocking the target genes related to tumor cell progression without causing noticeable damage to normal cells. Currently, efforts have been focused on improving the targeted therapy aspects regarding the encouraging outcomes in cancer treatment and the quality of life of patients. Treatment with targeted therapy for NSCLC is changing rapidly due to the pace of scientific research. Accordingly, this updated study aimed to discuss the tumor target antigens comprehensively and targeted therapy-related agents in NSCLC. The current study also summarized the available clinical trial studies for NSCLC patients.

## Introduction

Lung cancer accounts for about 13% of all cancers and is the number one cause of cancer-related death worldwide which leads to more deaths than colorectal, breast, brain, and prostate cancers [1, 2]. In 2022, the American Cancer Society estimated 236,740 new cases and 130,180 deaths from lung cancer in the United States. The prognosis of those with advanced disease, such as stage IIIB or stage IV, is poor and less than 5% [3]. Only about 15% of patients are diagnosed with early-stage disease, and most (84%) are in an advanced stage at the time of diagnosis. Altogether, Non-small cell lung cancer (NSCLC) patients have a poor prognosis and low 5-year overall survival (OS), approximately 17.4% [4–6].

NSCLC is the most common type of lung cancer, accounting for approximately 85% of the cases. The three most common forms of NSCLC are adenocarcinoma, squamous cell carcinoma as well as large cell carcinoma [7–9]. Common cancer treatment approaches include surgery, chemotherapy, and radiation therapy. Recent advances in science have led to the emergence of newer and more efficient methods, such as immunotherapy and target therapy, in treating various diseases, including infections, cancers, autoimmunities, and other disorders [10–12]. Over the past decade, immunotherapy and targeted therapy, along with other treatments, indicated successful outcomes in treating advanced lung cancer, especially NSCLC. They improved the overall survival (OS) of patients. This is predominantly due to the availability of biomarkers to select patients for targeted and immunotherapy-based treatments. In other words, treatments are shifting toward newer targeted and small molecule therapies to improve outcomes among NSCLC patients. Indeed, investigation of the human genome has permitted more efficient identification of gene alterations that are potential "targets" for therapy [13–15]. However, targeted therapy is indicated for those with distant metastases and stage IV disease.

Our main goal in the present study is to investigate categorized targeted therapy strategies in treating NSCLC. In the beginning, we give a general explanation of the NSCLC common treatments and then continue our study by focusing on the targeted therapy of NSCLC, which be discussed separately in the VEGF, KRAS, EGFR, ALK, ROS1, BRAF, RET, MET, NTRK, HER2, HER3, PI3K/AKT/mTORC, PD1, and CTLA-4 related sections.

## Treatment approaches for NSCLC

The treatment choices for NSCLC are based mainly on factors such as the tumor grade, size, and location, lymph node status, the patient’s overall health, and lung function. Surgery, chemotherapy, radiation therapy, and targeted therapy are the approved treatment modalities for NSCLC [16]. As a standard therapy approach, Stage 0 NSCLC is generally curable by surgery with no chemotherapy or radiation therapy. In addition, laser therapy, photodynamic therapy, or brachytherapy may be substituted for surgery. For stage I NSCLC, surgery is the main choice, and radiation therapy or adjuvant chemotherapy after surgery may lower the cancer recurrence. In stage II NSCLC, cancer removal may be followed by adjuvant chemotherapy, radiation, and immunotherapy. The initial treatment for stage IIIA NSCLC may comprise some combination of chemotherapy, radiation therapy, and/or surgery accompanied by immunotherapy. For people whose cancer cells have specific mutations in the *EGFR* gene, adjuvant therapy with the targeted drug osimertinib might be an option at some point additionally. Stage IIIB can’t be entirely removed by surgery, but chemoradiotherapy and immunotherapy are helpful. Stage IVA or IVB can be tough to cure, and treatments such as surgery, photodynamic therapy, laser therapy, radiation therapy, chemotherapy, immunotherapy, and targeted therapy may help by relieving symptoms. For stage IVB cancers that have metastasized all over the body, before any treatments, the cancer cells will be tested for certain gene mutations, including the *VEGF, KRAS, EGFR, ALK, ROS1, BRAF, RET, MET, NTRK* genes. If one of these genes is mutated, your first treatment will likely be a targeted therapy drug. Identifying gene mutations or some protein expressions in lung cancer has led to the development of molecularly targeted therapy to progress the survival of patients with advanced disease [17].

## Targeted drug therapy

**Fig. 1:**
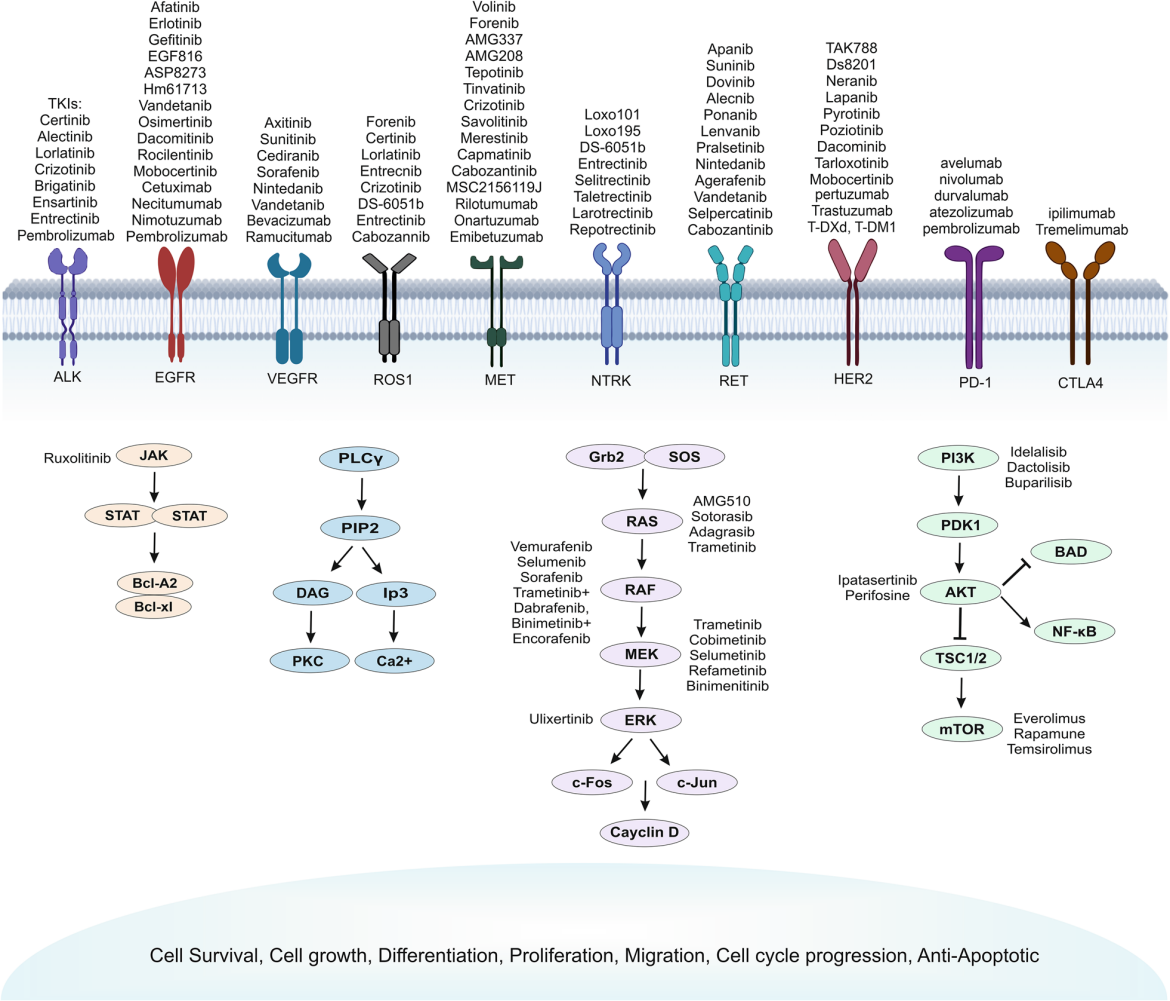
Target genes and drugs in NSCLC. Various targeted therapy drugs have been summarized that target different key related genes and their signaling pathways in NSCLC

Cancer cells have modifications in their genes or proteins that make them different from normal cells. Consequently, they can develop faster and sometimes could spread. Targeted cancer therapy works by those differences and targets cancer's specific genes and proteins that contribute to cancer growth and survival. So, it chunks the growth and spread of cancer cells, which confines the damage to healthy cells. Targeted drugs are often used for advanced lung cancers when conventional drugs don't affect them, mainly because they have different side effects. Many drugs targeting these pathways have been developed, and there are numerous FDA-approved targeted agents [5]. Today, inhibitors of EGFR, ALK, PI3K/AKT/mTOR, RAS-MAPK, RET, MET, BRAF, and NTRK/ROS1, as well as PD1 and CTLA4 molecules, are available for NSCLC, and many of these are now typical of care for selected patients (Fig. 1). In other words, to find the most effective treatment, personalized cancer therapy should be done by identifying the status of target genes and proteins in the patient by standard tests [5].

Despite these new therapeutic options for patients with advanced NSCLC, there continue to be significant challenges as resistance development and disease progression occur in most of these patients [18]. Nevertheless, while target therapy in NSCLC has provided disease control, the tumors inevitably develop drug resistance. Understanding resistance mechanisms and developing combinational therapies are essential for improving treatment outcomes.

### Vascular endothelial growth factor (VEGF)

Numerous studies demonstrated the efficient effect of small-molecule inhibitors of the Vascular Endothelial Growth Factor and its receptors (VEGF/VEGFR) in treating various malignancies, including NSCLC. There is two FDA-approved anti-VEGF pathway, Bevacizumab (Avastin/anti-VEGF-antibody) and Ramucirumab (Cyramza/anti-VEGFR antibody), for advanced NSCLC, which are used alone or in combination with chemotherapy. The VEGF pathway has been extensively studied and shown to play a critical role in angiogenesis [19]. Interestingly emerging evidence has demonstrated that VEGF may play a more comprehensive role in the pathogenesis of cancer than was previously thought, as it causes tumor-induced immunosuppression [20]. VEGFs can disrupt the maturation of dendritic cells (DCs) and other hematopoietic lineages and consequently down-regulate the antigen presentation process. Besides, it could enrich the infiltration of regulatory T (Treg) cells, tumor-associated macrophages (M2 macrophages), and Myeloid-Derived Suppressor Cells (MDSC) into the NSCLC niche [20–23]. In other words, blockade of the VEGF signaling leads to reversing these immune suppressive mechanisms, reducing the recruitment of suppressor cells into the tumor but increasing the infiltration of effector T cells.

Some studies revealed combination therapy of the other anti-angiogenic inhibitor or chemotherapy drug by anti-VEGF exerts a mostly synergistic anti-tumor effect. One study constructed a novel bispecific decoy receptor VEGFR-EGFR/Fc targeting the VEGF and EGF-like ligands in human NSCLC. The constructed dual-specific antibody inhibited tumor invasion, relocation, proliferation, and angiogenesis [24]. Interestingly, "Zhao" et al. revealed EGFR/VEGF inhibition therapy was transcendent to single EGFR inhibition, but not VEGF inhibition, for advanced NSCLC [25]. In addition, the combination of bevacizumab plus gemcitabine/cisplatin chemotherapy for advanced NSCLC had clinical efficacy and prolonged the long-term survival of patients [26]. Furthermore, it has been shown the combination of bevacizumab and erlotinib extended Progression-free survival (PFS) but increased the incidence of adverse events compared to their monotherapy in NSCLC patients [27].

Recently, some studies investigated the modulatory role of miRNA on the VEGF pathway. Wang et al. showed a decreased level of messenger RNA expressions of miR-199a and increased VEGF in NSCLC rat models. They declared that miR-199a prevents the proliferation of NSCLC cells via the targeted down-regulation of the *Hypoxia-inducible factor 1-*alpha /VEGF signaling pathway [28]. Another study showed that miR-214 targeted the inhibitor of Growth Family Member 4 in lung cancer cells and upregulated the HIF-1α pathway, leading to Matrix Metallopeptidase 2 and VEGF upregulation [29]. These studies indicate that targeted inhibition of the VEGF pathway by another mechanism could be investigated.

### KRAS

Kirsten Rat Sarcoma viral oncogene homolog (KRAS) gene is a gene that produces a protein that functions in the cell signaling systems that regulate cell division, maturation, and growth. It is the most mutated oncogene in human cancers, which hinders the development of effective drugs against KRAS [30, 31]. On that account, despite colossal endeavors that contributed to the development of drugs pointed at hindering KRAS or its signaling pathways, KRAS was historically considered undruggable. Nevertheless, several recent research that shows encouraging results initiating a new exciting era [32].

We now have a better understanding of the intricate interactions involving the RAS family of signaling proteins thanks to Ostrem, Shokat, and colleagues' discovery of the switch II pocket on the surface of the active and inactive forms of KRAS [33, 34]. According to all this research, two direct KRAS G12C (OFF) inhibitors, Sotorasib and Adagrasib, earned the breakthrough designation by the U.S. Food and Drug Administration (FDA) for the treatment of patients with KRAS G12C metastatic lung cancer and have shown promising results in phase I and II clinical trials (NCT04625647, NCT04933695, NCT04303780, NCT03785249) [35, 36].

Sotorasib (AMG510), developed by Amgen, is the first FDA-approved drug that irreversibly and selectively inactivates KRAS G12C using the interaction with a surface groove of the histidine 95 next to the cysteine 12 switch II pocket and keeps it in the inactive GDP-bound state. The compound AMG 510 has shown promise in phase I/II study (CodeBreak 100: NCT03600883) across solid tumors. In addition to CodeBreak 100, aiming for KRAS in NSCLC is a field of study. Recently, the phase 3 multicenter CodeBreak 200 clinical trial (NCT04303780) of a type of combined treatment using sotorasib and docetaxel has been used for patients with locally progressive and unresectable or metastatic NSCLC with KRAS G12C mutations [37–40]. Adagrasib (MRTX849) is another KRAS G12C (OFF) inhibitor that acts the same as sotorasib and binds KRAS G12C irreversibly and preferentially in its inactive GDP-bound state and also secures the switch II pocket. Adagrasib has been shown in clinical studies to have an anticancer effect against brain metastases and can reach the brain and cerebrospinal fluid. Phase I/II research evaluating the efficacy of adagrasib included participants with previously treated advanced or metastatic cancers, including NSCLC that carried the KRASG12C mutation. Adagrasib and docetaxel are also being compared in a phase III trial (KRYSTAL-12) for patients with recently treated KRASG12C-mutated NSCLC (NCT04685135), and numerous combination therapies with adagrasib are being developed [41–45].

### EGFR

The epidermal growth factor receptor (also known as ErbB1, HER1, or EGFR) is a member of a family of receptor tyrosine kinases that can activate a wide range of signaling pathways resulting in cell growth, differentiation, proliferation, and survival [46, 47]. After the ligand has been bound, the EGFR tyrosine kinase activates the receptor by homo- or heterodimerizing it and auto-phosphorylating tyrosine-rich cytoplasmic regions. This initiates the PIK3CA/AKT1/mTOR pathway and the RAS/RAF1/MAP2K1/MAPK1 kinases6, two major downstream intermediate pathways [48].

The EGFR mutation is one of the most significant genetic abnormalities in NSCLC patients among the rising driving oncogenes. Enhanced EGFR expression on cancerous cells (which is about 40–80% of NSCLCs), increased ligand synthesis by cancerous cells, and activating mutations of EGFR within cancerous cells are three primary factors that activate EGFR [49, 50].  Over 50% of adenocarcinomas and tumors from East Asians, never smokers, and women have these sorts of mutations, as do 10 to 20% of individuals with advanced NSCLC [51]. Complementary research demonstrated that activating mutations, not EGFR overexpression, were the main therapeutic target.

Around 20% of individuals with lung adenocarcinoma have EGFR mutations, including Exon 19 deletions (60%) and exon 21-point mutations (L858R missense replacements) at position 858 (35%), where leucine is changed to arginine, which cause fundamental activation of the receptor without ligand interaction [52, 53]. The first two drugs to target the tyrosine kinase domain of the EGFR were gefitinib and erlotinib. In both the phase I series and the subsequent phase II studies, these drugs showed encouraging action in NSCLC patients who had previously received chemotherapy. As a result, they have decided to approve the therapy for advanced NSCLC [54, 55]. Afatinib is another EGFR-TKI that is quite effective in treating advanced NSCLC patients with activating EGFR mutations in several clinical studies [56, 57].

In the first-line therapy approach, EGFR-TKIs are advised for NSCLC patients who have to activate EGFR mutations. Notably, chemotherapy is not necessarily the only treatment choice for individuals with wild-type EGFR NSCLC if there is no activating EGFR mutation. It is noteworthy that a sizable fraction of patients may still benefit clinically from EGFR-TKI therapy, even in those with wild-type EGFR NSCLC. There is ample proof that patients with wild-type EGFR NSCLC should not get EGFR-TKIs as their first-line therapy [58, 59]. A different approach to preventing EGFR activation and signaling is represented by monoclonal antibodies. In addition to forming antibody-receptor complexes that are endocytosed and destroyed, they can also completely prevent ligands from binding to the extracellular domain. The anti-EGFR mAbs cetuximab, necitumumab, panitumumab, and matuzumab are now accessible. Cetuximab and platinum doublet chemotherapy were used in two phase III trials, FLEX and BMS099, to treat advanced NSCLC [60, 61]. EGFR-TKIs considerably increase objective response rate (ORR), progression-free survival (PFS), and quality of life (QoL) compared to traditional chemotherapeutic regimens while exhibiting minor toxicity. The use of EGFR-TKIs has made significant advancements in the treatment of NSCLC, ushering in a new age of focused therapy and precision medication [62, 63].

### ALK

Anaplastic lymphoma kinase (ALK) is a different tyrosine kinase that has been thoroughly researched as a subject for TKI therapies. This 1620 amino acid transmembrane tyrosine kinase receptor is generated by the proto-oncogene ALK mostly expressed in the nervous system. ALK dimerizes and then auto-phosphorylates the intracellular kinase domain in response to ligand binding to its extracellular domain. This protein aids in regulating cellular development. Anaplastic large cell lymphoma, neuroblastoma, and non-small cell lung cancer are a few examples of cancers in which the anaplastic lymphoma kinase (ALK) gene may be altered. When activated in cancer, the cancer cells may develop and proliferate due to these ALK gene alterations. Gene fusions, chromosomal translocations, gene amplification or deregulation, and activating point mutations are the three main ways by which ALK signaling is triggered in tumor cells [64–67].

In 60 percent of anaplastic large cell lymphomas, a rare subtype of non-Hodgkin lymphomas, alk rearrangements were first discovered as a fusion to a section of the nucleophosmin (NPM) gene [68]. However, echinoderm microtubule-associated protein-like 4 (EML4) is the most common fusion component, and many EML4 breakpoints have been identified. Other uncommon fusion partners have also been identified; examples include TFG and KIF5B [69, 70]. About 2-7% of stage III or IV NSCLC patients and, more frequently, younger individuals, never or light smokers, which are more likely to develop brain metastases, have been shown to contain oncogenic fusion genes, including EML4 and ALK [71, 72]. Specific inhibitors, such as crizotinib, ceritinib, alectinib, etc., are highly beneficial in treating ALK-positive individuals, particularly those with non-small cell lung cancer [73].

The tyrosine kinases ALK and c-MET are specifically and powerfully inhibited by the tiny drug crizotinib. It is a first-generation c-MET inhibitor called PHA-66752 that was converted into an inhibitor of the 3-benzyloxy-2-aminopyridine series by leveraging the cocrystal structure of the inhibitor and c-MET to enhance active-site binding. In both first-line and second-line settings, crizotinib has been shown to increase progression-free survival (PFS) compared to chemotherapy [74, 75]. A second-generation ALK inhibitor, ceritinib, has been demonstrated to successfully treat advanced or metastatic ALK-positive NSCLC, including in patients who have already received crizotinib [76, 77]. The efficacy of these two ALK-targeted treatments may vary, according to recent research. Preclinical findings show that ceritinib is 20 times more effective against ALK than crizotinib. Additionally, it has been demonstrated that ceritinib is effective in individuals who have gained resistance to crizotinib, with remarkable tumor responses being seen in brain metastases [78, 79]. The evidence for the effectiveness of ceritinib combined with crizotinib in patients with advanced or metastatic NSCLC who have not taken crizotinib comes from five clinical trials: NCT01283516, NCT01685138, NCT00585195, NCT00932451, NCT00932893 [80, 81]. Expanding the therapy options and improving survival are effective targeted medications created against specific molecular subtypes of NSCLC, such as EGFR and ALK.

### C-ros oncogene 1 (ROS1)

ROS proto-oncogene 1 receptor tyrosine kinase (ROS1) rearrangement in NSCLC cell lines was first discovered in 2007 [82]. ROS1-rearranged NSCLC includes 1–2% of total NSCLC cases, all detected/diagnosed as adenocarcinomas. Although ROS1-positive prevalence was significantly higher in never-smoker young patients, the overall survival rate of ROS1-positive NSCLC indicated no significant variation compared with ROS1–negative [83]. There are FDA-approved ROS1 tyrosine kinase inhibitors (TKIs), such as crizotinib and entrectinib. Indeed, several other targeted TKIs are being developed or assessed in clinical trials.

#### Crizotinib (1st generation)

ALK and ROS1 share a 77% amino acid profile in their kinase domains [84]. Crizotinib efficacy in ALK-positive NSCLCs and ALK-RAS structural similarity suggested that ROS1 may be a potent therapeutic target for crizotinib. In 2016, crizotinib (Xalkori, PF-02341066) received approval from European Medicines Agency (EMA) and the United States Food and Drug Administration (FDA) for ROS1-rearranged NSCLC therapy [81]. crizotinib is the standard first-line treatment for ROS1-positive NSCLCs. Despite the significant efficacy of crizotinib against ROS1-rearranged NSCLCs, resistance to crizotinib leads to tumor relapse. The resistance to crizotinib is developed due to the acquired secondary point mutations, mainly attributed to the ROS1 G2032R mutation [85, 86]. This highlighted the pressing need to discover new potent ROS1-rearranged NSCLC-targeted therapies.

#### Entrectinib (2nd generation)

Entrectinib was first introduced in 2016 as a potent oral multi-target TKI against ROS1, ALK, and TRK kinases and gained FDA approval in ROS1-positive NSCLCs treatment [87]. A study disclosed in vitro and in vivo activities of entrectinib in NSCLC models [88]. Entrectinib showed substantial activity in ROS1-positive models. In addition, it has been reported that entrectinib was well penetrated through the blood–brain barrier (BBB) and induced intracranial activity, which interprets entrectinib potential efficacy in brain metastasis [88]. Multiple phase I/II cohort trials (STARTRK-1, STARTRK-2, and ALKA-372-001) of entrectinib have reported the efficient activity and manageable safety of entrectinib in ROS1 + NSCLC patients. The overall objective response rate was 77%, and the median duration of response was 24·6 months. Serious adverse events were observed in 11% of patients, and high-grade (grade 3 or 4) adverse events were reported in 34% of patients [89]. A recent study has compared the outcomes of two FDA-approved ROS1 inhibitors TKI, entrectinib, and crizotinib. The time-to-treatment discontinuation (TTD), PFS, and OS were variables of this comparison. These reports indicate further prolonged TTD using entrectinib (12.9 months) compared to crizotinib (8.8 months) [90].

#### Lorlatinib (3rd generation)

Lorlatinib (PF-06463922) is a selective third-generation tyrosine kinase inhibitor (TKI). It has been introduced as a ROS1/ALK inhibitor in crizotinib-resistant ROS1-positive NSCLC and glioblastoma. Lorlatinib showed high potency in NSCLCs with ROS1 G2032R mutation [91], which developed resistance to crizotinib [85, 86]. Oral lorlatinib showed a significantly higher ROS1 inhibition compared with crizotinib, alectinib, and ceritinib, which represents an encouraging alternative treatment in crizotinib-resistant ROS1-positive NSCLCs. In addition, lorlatinib improved CNS penetrance and reached a high level in cerebrospinal fluid (CSF), indicating that lorlatinib is a potential candidate for brain metastasis [91]. Phase I and II clinical trials in 2017–2019 investigated lorlatinib efficacy in ROS1- and ALK-rearranged NSCLC patients via addressing objective response rate (ORR) and median progression-free survival (PFS) [92, 93]. The clinical trials showed substantial activity in advanced ROS1-positive NSCLC patients. Lorlatinib showed a promising function in either crizotinib resistance or CNS metastasis. Additionally, there was no significant toxicity, and lorlatinib was well tolerated [92, 93]. Of note, lorlatinib showed in vitro anti-tumor activity against other crizotinib-resistant mutations such as L2026M, 66, 83 S1986Y/F, 66, and D2033N. Thus, Lorlatinib could be a potential alternative against advanced crizotinib resistance and metastasized NSCLCs.

### V-Raf murine sarcoma viral oncogene homolog B1 (BRAF)

BRAF is an oncogenic driver which accelerates the RAS–RAF–MEK–ERK pathway and induces cellular growth and hyper-proliferation [16]. BRAF gene mutations are present in 2–4% of NSCLC patients, of which approximately 50% of BRAF mutations have been detected as V600E [94, 95]. Non-V600E mutations, including G469A and D594G, account for 50% of total BRAF mutations [94–96]. Interestingly, in contrast with other rearrangements/drivers, non-V600E BRAF-driven cases are more associated with current/former smoking history [97, 98], while V600E BRAF mutant cases were commonly female or never smokers [98].

There are combined targeted therapies against V600 mutant melanoma that have achieved FDA approval. In 2014 the combined treatment of trametinib and dabrafenib, and later in 2020, the combination of atezolizumab with cobimetinib and vemurafenib was approved by FDA for patients with BRAF V600 mutation-positive melanoma [99]. The similarities in mutations and underlying mechanisms lead to several studies and clinical trials investigating these therapies for BRAF-positive NSCLC. BRAF mutation leads to the hyperactivity of the RAS–RAF–MEK–ERK axis, which exerts oncogenic features and drives hyper-proliferation [100]. Hence, blocking the members of the abovementioned pathway individually or together has shown encouraging effects against tumors.

#### Single treatment

Vemurafenib (PLX4032) was the first drug approved against BRAF-mutant cancer by the United States in 2011 [101]. Vemurafenib was first introduced 2008 as an oral selective inhibitor of oncogenic B-Raf kinase against melanoma harboring BRAF V600E mutation. Vemurafenib inhibits the activity of V600E BRAF mutation by blocking RAF/MEK/ERK pathway [102]. In 2015, vemurafenib administration by BRAF-positive NSCLC patients indicated a PFS of 7.3 months and an ORR of 42% [103]. In line with this study, in 2017, an open-label phase 2 study indicated that vemurafenib enhanced the PFS in previously untreated patients with V600 BRAF-driven NSCLC. Regarding toxicity, vemurafenib administration has been described as safe in these patients [104]. Dabrafenib selectively inhibits BRAF kinase and functions as an adenosine-triphosphate(ATP)-competitive inhibitor [105]. In 2016, an open-label phase 2 clinical trial study investigated the effect of dabrafenib in V600E BRAF-positive NSCLC cases. It has been reported that most patients had a rapid response to the treatment. Adenocarcinoma was controlled in 58% of patients, whereas the overall response was 33% [106]. This investigation was the first study (cohort A) of a series of three enrolled cohorts [106–108]. Cohorts B and C investigated the trametinib and dabrafenib combination, which will be mentioned below.

#### Adjuvant treatment/Co-administration

Dabrafenib + Trametinib (combined BRAF/MEK inhibition): Several clinical trials have investigated the combination of BRAF/MEK inhibition to develop a synergistic response. Planchard et al., in cohort B study, investigated the therapeutic outcome and toxicity of trametinib and dabrafenib combination for the first time in a phase 2 clinical trial [107]. This combined strategy was investigated in BRAF V600E-mutant metastatic NSCLC patients who were previously treated with platinum-based chemotherapy. The reported median PFS was longer than 9 months, and the overall response rate was more than 50%, which presents this combination as a promising candidate. Of note, in melanoma, intense adverse effects such as pyrexia, anemia, confusional state, decreased appetite, etc., were reported in more than half of the patients (56%), although the safety profile was manageable, and no treatment-related deaths were reported [107]. Later in 2017, in a cohort C study [108], Planchard et al. investigated the abovementioned combination therapy (trametinib plus dabrafenib) in patients with treatment-naïve BRAF V600E-mutant metastatic NSCLC. Thus, the response rate and toxicity are comparable in previously treated vs untreated patients. Due to the promising results of trametinib and dabrafenib combination therapy, in 2017, the FDA approved this treatment option for patients harboring BRAF V600E-positive metastatic NSCLC [109].

Trametinib + Vemurafenib: In 2015, a study compared the outcome in patients treated with trametinib (a MEK inhibitor) with or without vemurafenib. A single administration of vemurafenib affected V600E-BRAF, whereas trametinib affected both V600E-BRAF and non-V600E-BRAF. It has been reported that vemurafenib and trametinib co-administration enhanced BIM (pro-apoptotic protein) and apoptosis compared to single trametinib administration. Thus the combination of a BRAF inhibitor and a MEK inhibitor showed encouraging efficiency. Of note, this study also investigated the vemurafenib and erlotinib combination in BRAF-V600E cells, and they observed no synergistic effect [110].

### Rearranged during transfection (RET)

RET fusions have been reported in approximately 1% of NSCLC cases. Similar to the ROS1 fusions, most of the RET-positive population never included smokers and young patients [111]. The autophosphorylation of RET TKI domains drives Proto-Oncogene pathways. It activates downstream pathways, such as phosphatidylinositol 3-kinase (PI3K)/AKT, RAS/MAPK, c-Jun N-terminal kinase (JNK, and RAS/extracellular signal-regulated kinase (ERK), which are involved in the cellular growth, differentiation, and proliferation [112]. Additionally, it has been reported that RET rearrangements in advanced NSCLC patients are independently associated with an increased risk of brain metastases [113].

Both selective and non-selective RET inhibitors have been tested in clinical trials. While the reported outcomes were controversial, FDA has recently approved two selective RET inhibitors (pralsetinib and selpercatinib) as therapeutic options for metastatic NSCLC patients harboring RET fusions [114, 115].

#### Selective RET inhibitors

Pralsetinib (BLU-667): Pralsetinib is a selective tyrosine TKI, which potently targets and inhibits RET fusions. A multi-cohort, open-label, phase 1/2 study (ARROW) study (NCT03037385) tested the potential activity and safety of the pralsetinib in both previously treated and untreated patients with RET-altered metastatic NSCLC [116]. The maximum tolerated dose was determined once-daily 400 mg, based on a phase 1 study [117]. The response rate was reported based on the ORR. The ORR of previously-treated patients with platinum-based chemotherapy was 61%, of which 6% showed a complete response. While the ORR of treatment-naïve patients was 70%, and the complete response rate was 11%. These data suggested a better ORR in treatment-naïve; however, previously treated patients showed linger median duration of response in the periods of 6 and 12 months [116]. Although no treatment-related deaths were reported, several adverse effects were observed, including neutropenia, hypertension, and anemia.

Altogether, these findings introduce oral pralsetinib as a potent, well-tolerated treatment for RET-altered metastatic NSCLC patients [116]. The response to pralsetinib in RET fusion-positive NSCLC patients was also assessed based on the alterations/clearance of the level of the RET circulating tumor DNA (ctDNA) in blood samples. Interestingly RET ctDNA was rapidly decreased in almost all patients with every range of administered pralsetinib doses. After 8 weeks, RET ctDNA was not detectable in 81% of NSCLC patients, which is a rapid response [118]. A recruiting, randomized, open-label phase III trial compares pralsetinib and conventional first-line treatments based on the PFS of RET-altered NSCLC patients. The experimental arm will randomly receive pralsetinib. The squamous cell lung carcinoma patients of the control arm will receive platinum + gemcitabine, while non-squamous cell lung cancer patients of the control arm will receive platinum + pemetrexed ± pembrolizumab. This study aims to determine the appropriate choice for the first-line treatment [119].

Selpercatinib (LOXO-292): Selpercatinib (LOXO-292) is an oral, highly selective TKI inhibitor. It is an ATP-competitive, small-molecule RET inhibitor which has been developed to inhibit resistant or activating RET mutations. Compared to the multi-kinase inhibitors (MKIs), selpercatinib has shown remarkable activity in RET-inhibition and meaningful less toxicity in vitro and in vivo [120]. Clinically, selpercatinib presented potent activity in a patient with KIF5B-RET fusion-positive lung cancer. The alectinib-resistant patient had previously received treatments, including whole-brain radiotherapy, immunotherapy, and chemotherapy. It has been reported that selpercatinib dramatically reduced neurological [120]. A recent phase I/II study (LIBRETTO-001, NCT03157128) investigated the efficacy and toxicity of selpercatinib separately in two different cohort studies in previously treated and treatment-naïve patients with advanced RET fusion-positive NSCLC [121]. The objective response (partial or complete) was considered the primary endpoint. The PFS, duration of response, and safety were secondary endpoints in this study. Treatment-naïve showed an 85% accurate response, while the objective response of previously treated patients was 64%. In line with prior observations, selpercatinib significantly exerted intracranial activities against brain metastasis. The median CNS duration of response was 10.1 months, and the objective intracranial response was reported to be 91%. Selpercatinib showed intracranial responses, significantly rapid response, and persistent activity. Due to the insignificant/absent off-target activities, selpercatinib induced low-grade toxicity, and most of the drug-induced grade 3 toxicities were manageable via dose modifications. Thus, selpercatinib could be employed as a long-term RET-targeted therapy in patients with NSCLC [121].

Due to the promising reports of selective RET inhibitors, several phase 3 studies investigated novel agents to introduce the best treatment as the first-line treatment option. A phase III study (LIBRETTO-431, NCT04194944) is comparing standard first-line treatments with selpercatinib in RET-fusion-positive NSCLC patients, and this trial is in recruiting stage. In this study, the PFS of the control group who receive platinum-based chemotherapy (carboplatin or cisplatin) with or without pembrolizumab will be those who received selpercatinib [122]. In addition, this recruiting-stage trial aims to investigate the efficacy and safety of selpercatinib in combination with chemotherapy ± pembrolizumab.

#### Non-selective RET inhibitors

Non-selective RET inhibitors are MKIs, which target multiple kinases, have shown limited efficacy, and induced off-target toxicity. Non-selective RET inhibitors, including vandetanib, cabozantinib, and lenvatinib, were the first MKIs tested in RET-altered NSCLC patients. In a study in 2017, the highest total response rate (37%) and median PFS (3.6 months) were attributed to cabozantinib. The response rates of sunitinib and vandetanib were reported at 22% and 18%, respectively, although no complete response was observed. The median PFS of sunitinib and vandetanib were reported at 2.2 and 2.9 months, respectively [123]. Due to the limited efficacy and potentially high off-target toxicity of the multi-kinase inhibitors (MKIs), selective RET inhibitors appear to be more appropriate candidates. Resistance to both FDA-approved RET inhibitors is Inevitable, and G810C has been reported as the most resistant RET mutant [82].

Thus, further investigations are required to develop novel selective RET inhibitors and combined therapies. A phase I/II clinical trial (NCT04161391) aims to investigate TPX-0046 efficacy, safety, and tolerability in drug-resistant and naïve RET-altered tumor models with Solvent Front Mutations (SMF). TPX-0046 has shown potent anti-tumor activity in vitro and in vivo against various RET alterations, particularly against SFM-mediated resistance. In phase II, this trial will test TPX-0046 in treatment-naive or pretreated stage IV NSCLC patients to investigate the ORR [124].

### mesenchymal-epithelial transition (MET)

Mesenchymal-Epithelial Transition (MET) is a proto-oncogene belonging to the tyrosine kinase receptors family that was activated following the binding to its ligand, hepatocyte growth factor (HGF) [125]. Besides the physiological function of MET in cell proliferation [126], this proto-oncogene undergoes various alterations like mutations, amplifications, and overexpression, which could lead to malignant cells [127]. For instance, it has been reported that MET is overexpressed in 20% of NSCLC and amplified in 1–5% of NSCLC [128, 129]. On the other hand, it has been introduced as one of the critical processes in metastasis, which is needed to revert the mesenchymal phenotype to the epithelial phenotype for attaching the cancer cells to other tissues [130]. In this regard, it has been shown that this aberrant MET expression is correlated with a poor clinical prognosis of NSCLC [131]. Thus, targeting MET as a therapeutic option has been investigated in clinical trials.

Small molecule inhibitors of MET are divided into three main groups according to the binding site on MET structure [132]. type I and II prevent ATP binding and activating of the receptor (with the distinct binding site), while type III binds allosteric sites rather than the ATP-binding site [133]. In addition, type I of MET inhibitors include two subtypes; Ia (i.e., Crizotinib) and Ib (i.e., Capmatinib, Tepotinib, and Savolitinib), which Ib group is more specific to MET and has less off-target effects [134]. Of note, Crizotinib was first approved for the treatment of ALK-rearranged NSCLC and ROS1-rearranged NSCLC, but then it has been demonstrated that it could be recruited in NSCLC with MET amplification [135]. After Crizotinib FDA approval, Capmatinib and Tepotinib were approved for metastatic NSCLC in patients with MET exon 14 (METex14) skipping mutation [136], as one of the main mutations, which contributes to NSCLC independently [137]. Type II inhibitors, including Cabozantinib, Glesatinib, and Merestinib, not only bind to a hydrophobic pocket beside the ATP binding site of MET but also can block different kinases such as RON, AXL, VEGFR2, etc. Although Cabozantinib was approved for the treatment of advanced medullary thyroid carcinoma and advanced clear-cell renal-cell carcinoma, the studies on the efficacy and safety of Cabozantinib in MET-mutated NSCLC have shown promising results [138]. Moreover, Tivantinib, as a type-III inhibitor, was used in combination with Erlotinib, as an RGFR inhibitor, which revealed its beneficial effects on patients with NSCLC [139].

Moreover, there are other various strategies in the targeting of MET, which were included as immunotherapy. Regardless the expression of PD-L1 is remarkable in MET exon 14 NSCLC [140], and the immune checkpoint blockage strategy seems ineffective as monotherapy in patients with MET exon 14 NSCLC [141, 142]. Conversely, the combination of Cabozantinib with other anti-PD-L1 monoclonal antibodies in clinical trials revealed the safety and efficacy of this combination approach [143]. In addition to combination therapy, recruiting monoclonal antibodies as a tool for targeting the MET has been developed to disrupt the HGF/MET interaction [144, 145]. In this regard, different MET-specific monoclonal antibodies have been designed and studied, such as Onartuzumab, Telisotuzumab, and Amivantamab [138]. Amivantamab is a bi-specific monoclonal antibody targeting either EGFR or MET approved for patients with advanced metastatic NSCLC [146].Regardless of the progressions in MET targeting strategies and promising results, the tumor heterogeneity and selecting an appropriate population of patients benefiting from targeted therapy is challenging [147]. In addition, acquired resistance is another issue not only in MET targeting but also in all TKI approaches [148].

### Neurotrophic tropomyosin receptor kinase (NTRK)

The Neurotrophic tropomyosin receptor kinase (NTRK) involves three transmembrane receptor tyrosine kinases, TRKA, TRKB, and TRKC are responsible for the development and maturation of the central nervous system physiologically [1]. These tyrosine kinase receptors mediate three main signaling pathways; Ras/Raf/MAPK pathway, PI3K/Akt/mTOR pathway, and PLCc/PKC pathway are critical for cell proliferation and plasticity [2, 3]. Physiologically, the activation of these receptors depends on three ligands, including nerve growth factor (NGF), brain-derived neurotrophic factor (BDNF), and neurotrophin 3 (NT-3) [3, 4]. Otherwise, some genetic alterations in NTRK genes have been shown in malignancies, such as mutations, amplifications, splice variants, and deletions, which could lead to ligand-independent activation of downstream signaling pathways [5, 6]. The most prevalent alteration reported in numerous cancers is NTRK fusions (less than 1% of patients with NSCLC) which leads to the production of a chimeric protein with oncogenic activation [7]. Although it seems that NTRK fusions are just a minority cause of NSCLC, the high prevalence of NSCLC in the world made NTRK an interesting target for developing inhibitory drugs [8].

Regardless of NTRK inhibiting by unselective tyrosine kinase inhibitors (TKIs) such as crizotinib, cabozantinib, etc., which inhibit a range of targets, other more selective drugs have been developed [9]. Consequently, larotrectinib and entrectinib are two drugs that could get FDA approve after showing promising results in clinical trials. Entrectinib is an oral TKI with more selective activity against TRK, ROS1, and ALK. Although this drug is not just particular for TRK, according to the result of clinical trials, the ORR in patients with NTRK fusion-positive NSCLC was 70% with minimal tolerable adverse effects [10, 11]. Finally, FDA granted accelerated approval to entrectinib for adults with NTRK fusion-positive solid tumors. In addition, larotrectinib is a selective pan-TRK inhibitor whose efficacy and safety in NSCLC, and other cancers have been demonstrated [12, 13]. Accordingly, in a short time after clinical trials started, FDA approved it for the treatment of adult and pediatric patients with NTRK fusion-positive tumors. Interestingly, it has been reported that patients enrolled in clinical trials with brain metastases efficiently respond to the treatment with entrectinib and relatively with larotrectinib [10, 14].

Due to the acquired resistance mutations in tumor cells treated with the first generation of NTRK inhibitors which affect the durability of the drug, the second generation of inhibitors has been developed [15]. This new generation of inhibitors has a specific macrocyclic structure which could circumvent the on-target mutation of NTRKs [16]. In this regard, three drugs are under investigation; selitrectinib, taletrectinib, and repotrectinib. Although FDA just granted repotrectinib for Fast-Track designation in patients with advanced solid tumors harboring NTRK gene fusion [17], the results of selitrectinib case reports and phase I/II trials of taletrectinib suggest the efficacy and safety of these medications in the treatment of tumors [18, 19]. furthermore, to clarify the direct effect of these medications on patients with NSCLC, more studies are needed.

### Neurotrophic tropomyosin receptor kinase (NTRK)

The Neurotrophic tropomyosin receptor kinase (NTRK) involves three transmembrane receptor tyrosine kinases, TRKA, TRKB, and TRKC, which are responsible for the development and maturation of the central nervous system physiologically [149]. These tyrosine kinase receptors mediate three main signaling pathways; Ras/Raf/MAPK pathway, PI3K/Akt/mTOR pathway, and PLCc/PKC pathway, which are critical for cell proliferation and plasticity [150, 151]. Physiologically, the activation of these receptors depends on three ligands, including nerve growth factor (NGF), brain-derived neurotrophic factor (BDNF), and neurotrophin 3 (NT-3) [151, 152]. Otherwise, some genetic alterations in NTRK genes have been shown in malignancies, such as mutations, amplifications, splice variants, and deletions, which could lead to ligand-independent activation of downstream signaling pathways [153, 154]. The most prevalent alteration reported in numerous cancers is NTRK fusions (less than 1% of patients with NSCLC), which leads to the production of a chimeric protein with oncogenic activation [155]. Although it seems that NTRK fusions are just a minority cause of NSCLC, the high prevalence of NSCLC in the world made NTRK an interesting target for developing inhibitory drugs [156].

Regardless of NTRK inhibiting by unselective tyrosine kinase inhibitors (TKIs) such as crizotinib, cabozantinib, etc., which inhibit a range of targets, other more selective drugs have been developed [157]. Consequently, larotrectinib and entrectinib are two drugs that could get FDA approve after showing promising results in clinical trials. Entrectinib is an oral TKI with more selective activity against TRK, ROS1, and ALK. Although this drug is not just selective for TRK, according to the result of clinical trials, the ORR in patients with NTRK fusion-positive NSCLC was 70% with minimal tolerable adverse effects [158, 159]. Finally, FDA granted accelerated approval of entrectinib for adults with NTRK fusion-positive solid tumors. In addition, larotrectinib is a selective pan-TRK inhibitor whose efficacy and safety in NSCLC, and other cancers have been demonstrated [160, 161]. Accordingly, in a short time after clinical trials started, FDA approved it for the treatment of adult and pediatric patients with NTRK fusion-positive tumors. Interestingly, it has been reported that patients enrolled in clinical trials with brain metastases efficiently respond to the treatment with entrectinib and relatively with larotrectinib [158, 162].

Due to the acquired resistance mutations in tumor cells treated with the first generation of NTRK inhibitors, which affect the durability of the drug, the second generation of inhibitors has been developed [163]. This new generation of inhibitors has a specific macrocyclic structure, which could circumvent the on-target mutation of NTRKs [164]. In this regard, three drugs are under investigation, selitrectinib, taletrectinib, and repotrectinib. Although FDA just granted repotrectinib for Fast-Track designation in patients with advanced solid tumors harboring NTRK gene fusion [165], the results of selitrectinib case reports and phase I/II trials of taletrectinib suggest the efficacy and safety of these medications in the treatment of tumors [166, 167]. Furthermore, to clarify the direct effect of these medications on patients with NSCLC, more studies are needed.

### Human epidermal growth factor receptor 2 (HER2)

The human epidermal growth factor receptors are a series of receptor tyrosine kinase (RTK) which consist of 4 members; HER1-4 encoding by ErbB1-4 genes respectively [168]. HER2 is a transmembrane glycoprotein, and no exact ligand has been demonstrated for it [169]. Although activation of HER2 leads to several proliferative or apoptotic signaling pathways, including MAPK, PI3K/AKT, and JAK/STAT, its oncogenic and tumorigenic roles have been clearly illustrated in different cancers [170]. In this regard, three main genetic alterations have been demonstrated that could lead to NSCLC; mutation, overexpression, and amplification. Their frequency has been reported to be about 2–4%, 11–32%, and 2–23% respectively, in various studies [171, 172]. Nevertheless, the exact definition of HER2-positive lung cancer has not been addressed.

Targeting HER2 by various inhibitors such as pyrotinib, poziotinib, lapatinib afatinib, dacomitinib, and neratinib has been studied as a therapeutic option in patients with NSCLC, but unhopefully, none of them could yet get approved [173–176]. Despite the unsatisfactory results of various TKIs in treating NSCLC, some clinical trials showed promising results. For instance, in the treatment of NSCLC patients harboring HER2 exon 20 mutations with pyrotinib (as an oral pan-HER TKI), an ORR of 53.3% and a median PFS of 6.4 months with no extreme adverse events have been reported [177]. Moreover, a new small molecule TKI, mobocertinib, which selectively inhibits EGFR insertion and HER2 mutation, is under investigation with primary acceptable results [178]. In immunotherapy, monoclonal antibodies targeting HER2 have been investigated for the treatment of NSCLC. In this regard, two drugs, pertuzumab and trastuzumab, which have been approved for HER2 + breast cancer, have been used in clinical trials, but more studies are needed to confirm their therapeutic effect on NSCLC [179–181]. In addition, antibody–drug conjugations have been developed as an effective strategy for targeting HER2. T-DMI and T-DXd are two antibody–drug conjugates in which trastuzumab is attached to emantisine and deruxtecan, respectively. Both of these drugs have been implicated in clinical trials and showed promising results, which caused to T-DXd drug to get FDA grant for HER2-mutant NSCLC and gastric cancer [182, 183]. The main issue in recruiting HER2-targeting therapeutic approaches in treating NSCLC is detecting the proper population of patients sensitive to these medications. In other words, HER2 + NSCLC should be defined clearly for researchers and clinicians. Moreover, the lack of pharmacodynamic and pharmacokinetic information is another limitation for recruiting these medications on larger scales. Accordingly, more clinical and preclinical studies are needed to design the optimal guideline.

### PI3K/AKT/mTORC

The PI3K/Akt/mTOR signaling pathway has been considered one of the most commonly altered molecular pathways in NSCLC. EGFR and KRAS, the types of oncogenic drivers in NSCLC, can activate the PI3K/AKT/mTOR pathway, which enhances cancer cell proliferation, metabolism, and survival [184]. Besides, the RTKs are known as an entry point to PI3K/AKT/mTOR pathway activation. RTK mutation and amplification lead to ligand-independent signaling in NSCLC. Accordingly, targeting the PI3K/Akt/mTOR signaling pathway could suppress these signals [185].

PI3K is a family of intracellular lipid kinases classified into three classes based on structure and function. Each class showed distinct roles in signal transduction that regulated oncogenic transformation and tumor maintenance. However, somatic mutation and amplification of PI3K classes have been found in patients with NSCLC [186]. Activation mutations in PIK3CA (encoding the catalytic subunit PI3Kα) and alterations of the tumor suppressor phosphatase and tensin homolog (PTEN) are reported in squamous and non-squamous NSCLC. Preclinical and clinical studies suggested that targeting the PI3K pathway cloud regresses PIK3CA-mutant lung cancer. The growth of PI3K-dependent NSCLC cell lines could block through simultaneous inhibition of multiple PI3K pathway components, reducing the NSCLC progression [187, 188]. PI3K inhibitors are classified as pan-PI3K and selective PI3K inhibitors [184]. Several pan-PI3K inhibitors, including, Pilaralisib (XL-147), Buparlisib (BKM120), PX-866, and Pictilisib (GDC-0941), have been tested in clinical trial phases. The data associated with partial responses did not indicate significant improvement in PFS and OS [184]. Hence, the pan-class I PI3K inhibitor therapies have not shown enough efficacy. Several clinical trials are performed based on isoform-specific class I PI3K inhibitors. Of these, Alpelisib (BYL719) and Taselisib (GDC-0032) are potent PI3K inhibitors, which target the p110α isoform and the p110α, p110γ, and p110δ isoforms, respectively. They have been under evaluation in a phase II study of patients with advanced NSCLC [189, 190]. Other Selective PI3K inhibitors, including INK1117, GSK2636771, AZD8186, and SAR260301, have been investigated in clinical phase I trials [184].

On the other hand, upregulation of the Akt pathway is significant in NSCLC patients associated with increased mTOR [191]. Preclinical studies in NSCLC cell lines demonstrated that Akt activation leads to PTEN, EGFR or PIK3CA mutation, or HER2 amplification [192]. Therefore, targeted inhibition of Akt is an efficient therapeutic strategy, as many studies have characterized the extensive list of Akt pathway inhibitors in development. Perifosine is an alkyl phospholipid (APL) that blocks Akt translocation to the membrane, preventing Akt phosphorylation and activation, the best-tolerated treatment against NSCLC [193]. The evidence illustrated that using perifosine debilitates the translocation of GSK3β to the cell membrane and the consequent phosphorylation by kinases to Akt and ERK.

Moreover, perifosine therapy can inhibit rapamycin and significantly reduce the level of p-GSK3β and GSK3β in A549-RR cells. These results suggested that perifosine can counteract several survival pathways activated by mTORC1 inhibition, including GSK3β. However, recent studies found that a combination of perifosine and rapamycin can be more efficient than monotherapy, with every single reagent inhibiting cell growth by regulating the activity of GSK3β [194]. MK-2206 is another potent and selective inhibitor of AKT with anti-proliferative activity. Based on in vitro and in vivo results, the combined effects of MK-2206 with RTK inhibitors such as lapatinib and erlotinib can significantly exert tumor inhibitory activities [195]. Also phase II trial indicated that the combination of MK2206 and erlotinib met its primary endpoint in patients pretreated with erlotinib [196].

Meanwhile, significant proportions of NSCLC demonstrated upregulation of the mTOR pathway. mTOR complexes (mTORC1 and mTORC2) play an essential role in maintaining cellular homeostasis and growth [197]. Phosphorylation of p70-ribosomal protein S6 kinase 1 (p70-S6K1) and elongation initiation factor (EIF)-4E binding protein 1 (4E-BP1), are two significant substrates of mTORC1, regulate numerous processes such as cell growth, proliferation, migration, and invasion in NSCLC patients [198]. mTORC2 directly phosphorylates and activates Akt and Protein Kinase Cα (PKCα) [199]. Accordingly, mTOR inhibitors can be an attractive strategy to prevent the progression of advanced NSCLC. Ongoing clinical trials have been developed and investigated in preclinical and clinical studies, including Everolimus, Sirolimus, Temsirolimus, and Ridaforolimus. Everolimus is an mTOR inhibitor that explicitly targets mTORC1. Conservative evaluation indicated that side effects were of mild severity in most patients treated with Everolimus [200]. On the other hand, Combination therapy of Everolimus and EGFR inhibitors showed limited antitumor activity in NSCLC patients with a mutation in the PI3K-AKT-mTOR pathway [201]. Based on the phase I/II study, the combination of Sirolimus with Pemetrexed demonstrated potential activity and appeared to have no severe safety concerns [202]. Using Temsirolimus as a single targeted agent failed to meet its efficacy endpoint [203]. Although, the combination of Temsirolimus and Neratinib (HER2 inhibitor) showed a 19% response in patients with HER2-mutant lung cancers [204]. Ridaforolimus treatment showed improvement in PSF and trending for better OS in NSCLC patients with KRAS-mutant [205]. NSCLC involves a multistep process and is associated with several intracellular pathways and several genetic alterations. Therefore, using a single targeted agent may not be optimal [203]. Dual targeting has been developed by blocking both PI3K and mTOR. Cyclin D and FOXO forkhead transcription factors are important downstream targets of PI3K/Akt signaling. BEZ235 downregulated cyclin D1 and cyclin D3 expression in NSCLC through transcriptional repression and proteasome-mediated degradation, ultimately arresting the cell cycle at the G1 phase and inhibiting PI3K/Akt activity [203]. BEZ235 also inhibited mTOR signaling mediated by reduced cyclin D expression [206]. Additionally, XL765 and GDC-0980 were tested in a phase Ib trial that demonstrated an acceptable safety profile in NSCLC patients [207, 208].

### Targeting programmed death receptor 1 (PD-1)/ PD-L1 pathway

PD-1/PD-L1 signaling axis is prominent in regulating immune responses and maintaining self-tolerance or tissue integrity [209]. PD-1 is a negative costimulatory receptor expressed primarily on the surface of activated T cells, B cells, natural killer T cells, activated monocytes, and dendritic cells (DCs) [210]. PD-1 binds with its ligands, PD-L1 and PD-L2, which belong to the B7 family and can be expressed by tumor cells, normal cells, and immune cells [211]. However, PD-1-expressed T cells mediated to inhibit effective anti-tumor immune responses. PD-1/PD-L1 interaction inhibits the expression of T cell transcription factors such as GATA-3 and T-bet. It also inhibits CD8+ cytotoxic T lymphocyte (CTL) function, survival, and proliferation and induces apoptosis of tumor-infiltrating T cells. Besides, regulatory T cell (Tregs) differentiation and maintaining their suppressive function can mediate PD-L1 expression. Thereby, inhibition of PD-1/PD-L1 pathways can activate the anti-tumor activity mediated by both effector T cell activation and Treg inhibition [212].

Whereas the migration of immune cells to the tumor exhibit anti-tumor activity, over time, the tumor microenvironment favoring to becomes immunosuppressive, intending the emergence of tumor-promoting cells such as M2 macrophages, T regs, and myeloid-derived suppressor cells (MDSCs). Cancer cells escape from immune recognition and use immune-inhibitory mechanisms to evade the immune system's defenses. Immune editing is a dynamic process used to modulate the immune microenvironment and improve immune cell function so it can induce the alternative mechanisms of immune evasion [213–215]. Targeting the PD-1/PD-L1 checkpoint could regulate immune responses to eliminate NSCLC. Modulating the PD-1/PD-L1 pathway is currently under development as potential immunotherapies for patients with NSCLC. Several PD-1/PD-L1 antibodies are approved for the first- and second-line setting that improved efficacy and longer duration of response compared to other standard treatments and demonstrated manageable toxicity profiles [216]. Nivolumab is a fully human monoclonal antibody against PD-1 that was approved for treating patients with NSCLC. The patient treated with nivolumab in first-line monotherapy did not show more PFS than those who received platinum-based chemotherapy in a broad population with a PD-L1 expression level of 5% or more. Nivolumab showed a favorable safety profile as compared with chemotherapy [217]. Besides, based on the phase 3 study, nivolumab was correlated with a significant improvement in overall survival and response rate versus docetaxel in advanced non-squamous NSCLC [218]. Pembrolizumab, MK-3475, a humanized monoclonal IgG4 anti-PD-1 antibody, is another anti-PD1 showing robust anti-tumor activity and significantly improved PFS and OS than chemotherapy as first-line therapy for metastatic NSCLC with PD-L1 tumor proportion score of at least 50%. There was a lower incidence of treatment-related AEs than platinum-based chemotherapy, and did not show any evidence of increased toxicity during long-term follow-up [219]. The analyses demonstrated that pembrolizumab had a better ORR than nivolumab, but the difference in PFS was not significant between pembrolizumab and nivolumab in patients with recurrent or advanced NSCLC [220]. The evidence indicated that NSCLC patients with EGFR- or HER2-mutated and ALK-rearranged do not benefit from immunotherapy. Also, BRAF- and MET-mutated NSCLC are supposed to be as sensitive to anti-PD1/PD-L1 immunotherapy [221]. Although anti-PD1 monotherapy such as nivolumab and pembrolizumab had similar efficacy in older and younger patients with NSCLC, survival was significantly worse in patients with poor performance status (PS). However, an immune checkpoint inhibitor may be considered for NSCLC patients with poor PS harboring positive PD-L1 expression [222]. Other immune checkpoints were potential therapeutic targets, including programmed death receptor ligand 1 (PD-L1). Atezolizumab is a humanized IgG1 antagonist antibody to PD-L1 that blocks the interaction between the PD-L1 and PD-1 receptors activation complex. It is designed to impede inhibitory signals in T cells, with resultant tumor recognition by cytotoxic T cells. atezolizumab therapy improved the OS and ORR in patients with NSCLC expressing PD-L1 [223]. Cemiplimab monotherapy significantly enhanced OS and PFS versus chemotherapy in patients with advanced non-small-cell lung cancer with PD-L1 of at least 50% [224]. Moreover, durvalumab is a selective, high-affinity, engineered, human IgG1 monoclonal antibody that blocks the interaction between PD-L1 and PD-1, allowing T cells to recognize and kill tumor cells. Based on the real-world prospective study, durvalumab therapy had a safety profile and improved progression-free survival and OS among patients with stage III NSCLC [225, 226]. According to meta-analysis data, anti-PD-1 and anti-PD-L1 therapy had significantly longer OS and illustrated a lower ORR, higher 3–4 Grade AEs rate, and higher drug-related death than chemotherapy [227]. However, the Phase 3 trial demonstrated that Avelumab did not significantly improve OS versus docetaxel in patients with NSCLC with PD-L1 + tumors [228].

Additionally, Epigenetic mechanisms were used to modulate PD-L1 expression in cancer cells. miR-200c is one miRNA that directly binds to the 3′UTR of PD-L1 in NSCLC to inhibit PD-L1 expression [229]. miR-135 and miR-3127-5p positively regulate PD-L1 expression in NSCLC that indirectly induce PD-L1 expression by activating the PI3K-AKT-mTOR pathway [230, 231]. On the other hand, the methylation status of the PD-L1 promoter can use to predict the outcome of PD-1/PD-L1-targeted therapy. Anti-PD1 therapy may enhance drug resistance to anti-PD-1 immunotherapy Nivolumab in NSCLC patients through increasing PD-L1 promoter methylation and decreased PD-L1 expression [232].

### Cytotoxic T-lymphocyte-associated antigen-4 (CTLA-4)

CTLA-4 (Cytotoxic T-lymphocyte-associated antigen-4, CD152) is a type of immune checkpoint pathway that mediates negative regulation of T cell activation and preservation of self-tolerance. However, CTLA-4 expression is a potential prognostic and predictive biomarker in NSCLC patients. Evidence showed that the expression of CTLA-4 has a different prognostic effect in metastatic NSCLC lymph nodes versus primary tumors, which is mediated by phenotypical differences between the tumor microenvironments of lymph nodes and primary tumors [233]. CTAL-4 is considered a leader of the immune checkpoint inhibitors, which potentially suppressing autoreactive T cells at the initial stage of naive T-cell activation, typically in lymph nodes [234]. The inhibitory function of CTLA-4 was administered through several mechanisms, such as competition with CD28-positive costimulation for binding to their shared B7 ligands (CD80/CD86) [235]. CTLA-4 is considered the homolog of CD28 that shows a higher binding affinity for B7 than CD28. CTLA-4:B7 binding can produce an inhibitory signal through contraction of the stimulatory signals from CD28:B7 and TCR: MHC binding. However, CTLA-4:B7 inhibitory signals include direct inhibition at the TCR immune synapse and enhanced mobility of T cells, causing the reduced ability to interact with APCs [234].

Besides, the independent prognostic effect of CTLA-4 overexpression was indicated in NSCLC, a favorable impact of CTLA-4 overexpression on clinical outcomes. CTLA-4 overexpression can cause a worse prognosis due to an enhanced downregulation of T-cell activation. Indeed, it showed that CTLA-4 mediated negative signals into cancer cells, compared with the ones currently observed in T cells [236]. However, the permanent expression of CTLA-4 on cancer cells showed a critical role in cancer cell progression by producing inhibitory signals to weaken the immune response. Hence, targeting CTLA-4 is an attractive strategy for increasing immune efficacy against malignancies and improving the prognosis of tumor patients. Multiple preclinical and clinical trials were performed to test the antibodies targeting CTLA-4, including ipilimumab and tremelimumab [235]. Ipilimumab is a fully humanized IgG1κ mAb targeting CTLA4. Treatment with ipilimumab improved irPFS and mWHO-PFS and safety and tolerability without the severe impact of toxicities in patients with stage IV NSCLC [237]. Tremelimumab is another monoclonal immunoglobulin G2 antibody against CTLA-4 that enhances immune function via preventing normal downregulation of T cells and prolonging T-cell action [238]. Tremelimumab indicated a safety profile and improved the durability of objective responses than standard chemotherapy in patients with advanced cancer such as NSCLC [239]. Clinical trial results illustrated that anti-CTLA4 mAbs lead to acute immune activation, which increases CD8+ CTL infiltrates in the tumor and may produce transient autoimmune manifestations. Corticosteroid therapy has been planned with CTLA-4 blockage to prevent the adverse event. Besides, a combination of CTLA-4 with GM-CSF-expressing tumor cell vaccine can be therapeutically effective; it targets prominent regulatory pathways of the immune system and modifies the immune response [239].

However, CTLA-4 blockade was suggested as a regulatory checkpoint for therapeutic development. Dual checkpoint therapy may be more efficient and reduce the adverts event of monotherapy with anti-CTLA-4 therapy. Also, PD-L1 expression and tumor mutational burden are potential biomarkers to respond to the combination approaches in these treatments. Based on First-line treatment, a combination of nivolumab plus ipilimumab improved PFS compared with chemotherapy in NSCLC patients with high tumor mutational burden. In comparison, patients with a low tumor mutational burden showed similar PFS in the nivolumab-plus-ipilimumab and chemotherapy groups. Dual therapy exhibited better efficacy than monotherapy in patients with a high tumor mutational burden [240]. Combination therapy tremelimumab with durvalumab has demonstrated clinical activity in advance-NSCLC patients. Based on a phase 3 randomized clinical trial, durvalumab plus tremelimumab did not statistically significantly enhance OS or PFS versus chemotherapy in patients with PD-L1 tumor proportion score ≥ 25%, while there was improved OS or PFS in durvalumab plus tremelimumab than chemotherapy in patients with 25% of tumor cells expressing PD-L1. Also, the dual checkpoint inhibition showed optimal benefit in OS and enhanced PFS. In addition, dual immunotherapy showed a high rate of TRAEs that was causing to discontinuation than durvalumab or chemotherapy [241].

## FDA approved Clinical trials for NSCLC-targeted therapy

Clinical and preclinical studies indicated the efficacy and safety of multiple therapies for NSCLC patients with different aberrations, including EGFR exon 19 deletions or exon 21 (L858R) mutations, RET fusion-positive metastatic, ALK genomic tumor aberrations, EGFR exon 20 insertions. However, based on clinical trial results, several targeted therapies were approved by U.S. Food and Drug Administration (FDA) for NSCLC (Table 1). The receptor tyrosine kinase inhibitors, including erlotinib [242], gefitinib [243], osimertinib [244], crizotinib [245], lorlatinib [246], tepotinib [247, 248] received first approval for treating patients with EGFR mutation, acquired EGFR TKI resistance, ALK-positive, METex14 skipping alterations, respectively. In addition, immune checkpoint inhibitors such as atezolizumab [249], cemiplimab [250], and nivolumab plus ipilimumab [251] received FDA approval for treating patients with high PD-L1 expression and no EGFR or ALK genomic tumor aberrations. Moreover, Amivantamab, an intravenously administered bispecific antibody targeting EGFR and c-MET, received its first FDA approval for treating NSCLC with EGFR Exon 20 insertion mutations [146]. Based on findings from the CHRYSALIS clinical trial (NCT02609776), Amivantamab showed robust and durable responses with tolerable safety, which an ORR rate of 40%, a median PFS of 8.3 months, and a median OS of 22.8 months [252]. Unfortunately, according to recent evidence, the disease's progression and no treatment response have been shown in patients [253]. Recently, preclinical investigation supported the clinical development of mobocertinib for treating EGFRex20ins-mutated NSCLC [254].

**Table 1:** The summarized targeted therapy studies in NSCLC

Year	Drug	Type of inhibition	Patient	Company	Recommended dose	Outcomes	Refs.
2011	Crizotinib	A small molecule TKI	NSCLC with ALK-positive	Pfizer	250 mg orally twice daily	(ORR) of 61% (95% CI 52%–70%), PFS of 7.7	[242]
2013	Erlotinib (tarceva)	EGFR TKI	NSCLC with EGFR exon 19 deletions or exon 21 (L858R) substitution mutations	Astellas Pharma Inc	150 mg/day	ORR: 65%, PFS: 10.4 months, OS: 22.9 months	[239]
2015	Gefitinib	Selective small-molecule EGFR TKI	NSCLC with EGFR exon 19 deletions or exon 21 (L858R) substitution mutations	AstraZeneca	250 mg daily	ORR: 50% (95%CI 41–59), DoR: 6.0 months	[240]
2015	Osimertinib (TagrissoTM, AZD9291)	Third-generation EGFR TKI	NSCLC patients with acquired EGFR TKI resistance	AstraZeneca	80 or 160 mg/day for a median of 260 and 171 days, respectively	ORR: 70%	[241]
2018	Lorlatinib (lorbrena^®^)	ATP-competitive small molecule inhibitor of TKI	ALK-positive NSCLC	Pfizer	100 mg once daily	ROS1‑Positive NSCLC: ORR: 36.2% (95%CI 22.7–51.5), intracranial ORR: 56.0% (95%CI 34.9–75.6), PFS: 9.9 (95%CI 5.5, 21.0)	[243]
2020	Ramucirumab plus erlotinib	Anti-VEGFR2 agent	NSCLC with epidermal growth factor receptor (EGFR) exon 19 deletions or exon 21 (L858R) mutations	Eli Lilly	10 mg/kg every 2 weeks	ORR: 76%, PFS: 19.4 months	[252]
2020	Pralsetinib (GAVRETO™)	RET inhibitor	Patients with RET fusion-positive metastatic NSCLC	Blueprint Medicines Corporation	400 mg orally once daily		[253]
2020	Nivolumab ( OPDIVO) plus ipilimumab ( YERVOY)	Anti-PD1, Anti-CTLA-4	NSCLC with no epidermal growth factor receptor (EGFR) or anaplastic lymphoma kinase (ALK) genomic tumor aberrations	Bristol-Myers Squibb Co	360 mg nivolumab every 3 weeks with ipilimumab 1 mg/kg every 6 weeks	ORR: 38% (95% CI 33, 43) PFS: 6.8 months (95% CI 5.6, 7.7) OS: 14.1 months (95% CI 13.2, 16.2)	[248]
2020	brigatinib (ALUNBRIG)	Tyrosine kinase inhibitor	Anaplastic lymphoma kinase (ALK)-positive metastatic NSCLC	ARIAD Pharmaceuticals Inc	90 mg orally once daily for the first 7 days; then increase to 180 mg orally once daily. Brigatinib may be taken with or without food	ORR: 74% (95% CI 66, 81) PFS: 24 months (95% CI 18.5, NE)	[254]
2020	Atezolizumab (TECENTRIQ^®^)	Anti-PD-L1	NSCLC with high PD-L1 expression and no EGFR or ALK genomic tumor aberrations	Genentech Inc	840 mg every 2 weeks, 1200 mg every 3 weeks, or 1680 mg every 4 weeks, administered intravenously over 60 min	ORR: 38% (95% CI 29, 48), PFS: 8.1 months (95% CI 6.8, 11.0), OS: 20.2 months (95% CI 16.5, NE)	[246]
2020	Tepotinib (Tepmetko^™^)	MET TKI	Advanced or recurrent NSCLC with METex14 skipping alterations	Merck	500 mg once daily	PFS: 9.5–12.2 months	[244, 245]
2020	Capmatinib ( Tabrecta^™^)	Oral MET inhibitor	NSCLC with MET exon 14 skipping mutations	Novartis	400 mg orally twice daily	ORR: 68% (95% CI 48, 84)	[255]
2020	Selpercatinib (RETEVMO	ATP-competitive, highly selective small-molecule inhibitor of RET kinase	metastatic RET fusion-positive NSCLC	Eli Lilly	120 mg for patients less than 50 kg, and 160 mg for those 50 kg or greater	ORR: 85% (95% CI 70%, 94%)	[256]
2021	Sotorasib (LUMAKRAS^™^)	RAS GTPase family inhibitor	NSCLC KRA mutations	Amgen	960 mg taken orally once daily (with or without food)	ORR: 35% DCR: 91% median DOR: 10.9 months PFS: 6.3 months	[35, 257]
2021	Cemiplimab-rwlc (Libtayo^®^)	Anti-PD-L1	NSCLC with high PD-L1 expression (Tumor Proportion Score [TPS] > 50%) and no EGFR, ALK or ROS1 aberrations	Regeneron Pharmaceuticals, Inc	350 mg every 3 weeks, intravenously over 30 min	ORR: 37% (95% CI 32, 42) PFS: 6.2 months (4.5, 8.3) OS: 22.1 months	[247]
2021	Amivantamab-vmjw (Rybrevant^®^)	Monoclonal antibody directed against EGFR and MET	NSCLC with EGFR exon 20 insertions	Janssen Biotech	1050 mg (if bodyweight was < 80 kg) or 1400 mg (if bodyweight was ≥ 80 kg) once weekly for 4 weeks	ORR: 40% (95% CI 29–51%), DOR: 11.1 months (95% CI 6.9 to not estimable), PFS: 8.3 month OS: 22.8 months	[143, 258]

*NSCLC* non-small cell lung cancer, *EGFR* epidermal growth factor receptor, *TKI* tyrosine kinase inhibitor, *RET* rearranged during transfection, *MET* mesenchymal-epithelial transition factor, *ORR* objective response rate, *DoR* duration of response, *PFS* progression-free survival, *OS* overall survival

## Combination therapy

In order to overcome the limitations of target therapy and increase its efficiency, using this treatment in combination with other cancer treatment methods can be more effective. There are multiple examples of combined treatment of target therapy with other common treatments used for NSCLC patients, especially chemotherapy. Various studies have been conducted in this field, which can help to increase the efficiency of target therapy and overcome the limitations of this treatment method, especially drug resistance [255]. Extensive research has been conducted on the comparison of targeted therapy drugs with chemotherapy agents, including cetuximab, an anti-EGF-R monoclonal antibody, with cisplatin and vinorelbine [61], Bevacizumab, a mAb targeting the VEGF in combination with carboplatin and paclitaxel [256], and Figitumumab, a mAb targeting insulin-like growth factor type 1 receptor (IGF-1R), along with paclitaxel and carboplatin or gemcitabine/cisplatinum regimen [257]. On average, the results of all studies were similar. They showed that the combination therapy was effective and safe, and the group receiving these treatments had higher response rates, median progression-free survival, and overall survival. The combination of a c-MET inhibitor and the EGF-R inhibitor erlotinib was used compared to using either inhibitor alone in the NSCLC tumor xenograft mice model and indicated a significant anti-tumor function [258]. Vandetanib is a multi-target TKI that selectively targets VEGF-R, EGF-R, and RET tyrosine kinase activity. Similar to mentioned combination therapies, Vandetanib and docetaxel, exhibited promising outcomes with increased progression-free survival (PFS). It also showed that Vandetanib controlled lung cancer symptoms longer than chemotherapy alone [259, 260]. Promising results have also been reported from the conducted and ongoing trials on the effect of combination therapy sunitinib and erlotinib, as well as sunitinib along with platinum-based chemotherapy. Sunitinib is a multitargeted TKI with antiangiogenic and antitumor activities that inhibits VEGFR, PDGFR, KIT, RET, and Flt-3 [261]. Similar studies have reported promising results of combination therapy of everolimus, the mammalian target of rapamycin (mTOR) inhibitor with chemotherapy or combination with chemotherapy/gefitinib regimen, including higher survival, good response to therapy, and improvement in disease symptoms [200, 262]. Also, target therapy in combination with radiotherapy or combined chemotherapy/radiotherapy has resulted in promising results of improved survival and response to treatment. Examples are cetuximab, an EGFR inhibitor, along with radiotherapy and chemotherapy in advanced lung cancer [263, 264], as well as Gefitinib and erlotinib integrated into chemoradiation [265, 266].

## Limitations of NSCLC targeted therapy

The widespread application of targeted therapies for advanced NSCLC to potentially cure disease, is hindered by several significant challenges. These include drug resistance, toxicity, and the high cost of these agents, which can limit their accessibility to all NSCLC patients.

### Tumor heterogeneity and Lack of validated biomarkers

Another limitation of targeted therapy in NSCLC is the lack of validated biomarkers to predict response to treatment due to tumor heterogeneity [255]. While molecular testing is used to identify patients who may benefit from targeted therapy, not all patients with a particular molecular abnormality will respond to treatment. Additionally, some molecular abnormalities may be present in only a subset of cancer cells within a tumor, leading to incomplete targeting and potential resistance [267, 268]. Most targeted therapies have been developed for advanced-stage NSCLC patients who have already developed molecular abnormalities that can be targeted. However, early-stage NSCLC patients may not have these molecular abnormalities, and there may not be targeted therapies available for their specific subtype of NSCLC [269, 270]. This can make it challenging to identify which patients should receive targeted therapy and which should receive other types of treatment [255, 269, 271]. Additionally, even when molecular abnormalities are identified, there is often a lack of evidence on the optimal treatment approach, including which targeted therapy to use and the best sequencing of therapies.

### Drug resistance

Resistance is a major limitation of targeted therapy in NSCLC, where cancer cells that were initially sensitive to treatment eventually become resistant and continue to grow and spread. Resistance can occur due to various mechanisms, including genetic mutations, alterations in gene expression, and changes in the tumor microenvironment. Sometimes, changes like mutations and amplifications occur in the target gene that allows cells to continue growing even when an inhibitor is present [255]. Targeted therapies can create selective pressure, which causes abnormalities that activate the driver oncogene. Malignant tumors with specific abnormalities initially respond well to selective inhibitors. However, resistance develops over time, often due to genetic aberrations in the target gene. For example, secondary mutations like EGFR C797S have been seen after osimertinib therapy, while EGFR T790M mutations can lead to resistance with erlotinib or gefitinib [272, 273]. Additionally, crizotinib has been shown to lead to on-target resistance in patients with ALK and ROS-1 mutations (L1196M and L2026M, respectively) [274]. These genetic changes and subsequent resistance are significant limitations to the effectiveness of targeted therapies in NSCLC. In NSCLC cells, acquired resistance to gefitinib or osimertinib has shown EMT characteristics, such as a decrease in E-cadherin and an increase in vimentin and stemness, without any secondary EGFR mutations [275]. AXL is implicated in the pathogenesis of EMT, and AXL inhibitors may restore sensitivity to erlotinib in mesenchymal EGFR-mutant cells [276]. While these inhibitors have shown significant efficacy in some patients with NSCLC, resistance can develop due to a variety of mechanisms, including downregulation of antigen presentation, upregulation of alternative immune checkpoints, and alterations in the tumor microenvironment.

### Toxicity

Although short-term exposure to targeted therapy drugs is generally manageable, their prolonged use for chronic administration often results in significant toxic side effects. Targeted therapies were initially considered less harmful because they specifically target cancer cells. However, both "on-target" and "off-target" effects of these therapies can lead to toxicity. Also, some targeted therapies can cause serious adverse events such as liver or kidney toxicity, blood clots, and gastrointestinal problems [255]. These side effects can limit the dose and duration of therapy, reducing the effectiveness of the treatment. The on-target toxicity occurs when a drug designed to inhibit cancer-specific targets also inhibits a fixed set of proteins in normal cells, leading to toxicity by inhibiting the signaling pathway. This can result in a range of toxic side effects, including hyperglycemia with PI3K inhibition, hypertension with vascular endothelial growth factor inhibition, and skin rash with EGFR inhibition [277, 278]. Off-target toxicity occurs when a drug blocks a non-target protein, leading to harmful side effects. Unlike on-target toxicity, off-target toxicity is specific to a particular drug and does not exhibit a class effect. For example, osimertinib is associated with cardiac toxicity, such as heart failure, left ventricular dysfunction, conduction abnormalities, and myocardial injury, while gefitinib is not [279, 280].

## Conclusions and future perspective

Over the last decade, the progress of molecular pathology has improved our knowledge of the pathophysiology and heterogeneity of NSCLC, resulting in significant evolution in treating patients with more advanced and effective methods. Target therapy and immunotherapy have opened a new and promising perspective in treating lung cancer by achieving successful outcomes. Several signaling pathways and specific oncogenic driver mutations have been identified that cause malignant alterations. New targets, such as microRNAs, VEGF, ALK, MET, HER2, and immune checkpoint inhibitors (PD-1 and CTLA4), as well as signaling pathways like PI3K/AKT/mTOR, RAS/MAPK, and NTRK/ROS1 pathways, are constantly discovered in addition to the epidermal growth factor receptor (EGFR), driving the emergence of new treatments. These factors and pathways have been the target of numerous medicines that have demonstrated clinical efficacy. Currently, some of these drugs, including EGFR inhibitors (gefitinib and erlotinib), PI3K/AKT/mTOR inhibitors (everolimus), and NTRK/ROS1 inhibitors (entrectinib) are being used as first-line therapies instead of chemotherapy. Interestingly, many NSCLC patients hopefully respond to checkpoint inhibitors, such as the anti-PD1 antibodies (nivolumab and pembrolizumab). Additionally, some research has indicated that certain targeted treatments combined with immunotherapies are effective in NSCLC. Drug resistance in the tumors is inevitable even though target therapy for NSCLC has controlled the disease. For improving treatment effectiveness, combination drug development and understanding resistance mechanisms are essential. Up to now, numerous clinical trials evaluating targeted therapy and immunotherapy agents are now being conducted, and the outcomes thus far are encouraging. The findings of trials will shed light on defining targeted therapy involvement in the treatment of NSCLC and clarify the roles of immune-based monotherapies, combined immunotherapies, and targeted therapy-immunotherapy combinations. Targeted therapy may ultimately change the way that lung cancer is treated for patients with few therapeutic options. Clinical research is still being done to discover the predictive factors responding to targeted therapies. Improved molecular biomarker knowledge and developed combined therapies might provide the most effective cure. Proper patient selection using predictive biomarkers will be crucial for theranostics and truly customized oncological treatment in the future to maximize the limited resources available and decrease vulnerability. Future gains in patient survival are anticipated if resistance is addressed, the suitable inhibitor or combination of inhibitors is used, and side effects minimal.

## Data Availability

Not applicable.
